# Interleukin-15 (IL-15) and IL-15 receptor alpha fusion protein enhances antitumor activity of myxoma virus

**DOI:** 10.1186/2051-1426-1-S1-P137

**Published:** 2013-11-07

**Authors:** Vesna Tosic, Diana L Thomas, David M Kranz, Jia Liu, Grant McFadden, Amy L MacNeill, Edward J Roy

**Affiliations:** 1 MIP, University of Illinois, Urbana, IL, USA; 2 Neuroscience, University of Illinois, Urbana, IL, USA; 3 Biochemistry, University of Illinois, Urbana, IL, USA; 4 Pathobiology at College of Veterinary Medicine, University of Illinois, Urbana, IL, USA; 5 Molecular Genetics and Microbiology, University of Florida, Gainesville, FL, USA

## 

Myxoma virus, a rabbit poxvirus, can efficiently infect various classes of mouse and human cancer cells. It is a strict rabbit-specific pathogen, safe to use in all non-rabbit hosts tested including mice and humans. Recombinant viruses were previously engineered to express tdTomatoRed fluorescent protein (vMyx-tdTr) and mouse interleukin-15 (vMyx-IL15-tdTr). IL15 is an immuno-modulatory cytokine with a great potential for stimulating T lymphocytes and NK cells. It has been shown that coexpression of IL15 with the α subunit of IL15 receptor (IL15Rα) greatly enhances IL15 stability and bioavailability. Our previous studies have shown that earlier generation recombinant myxoma viruses (vMyx-tdTr and vMyx-IL15-tdTr) selectively infected tumors, but had limited therapeutic effect in vivo. In order to use myxoma virus as a vehicle to deliver immuno-stimulatory cytokine to tumors, we engineered a new recombinant myxoma virus (vMyx-IL15Ra-tdTr), which expresses IL15Rα-IL15 fusion protein and tdTomatoRed fluorescent protein. Multi-step growth curves show productive infection of various cancer cell lines tested. Melanoma (B16-F10 and B16.SIY) and glioma (GL261 and GL261.SIY) cell lines are permissive to myxoma infection. RK-13 cells infected with vMyx-IL15Ra-tdTr (MOI=5) express and secrete the IL15Rα-IL15 fusion protein. Functional activity of the secreted fusion protein in vitro is confirmed by stimulating proliferation of the cytokine-dependent CTLL-2 cells. In vivo experiments, in which RAG-/- mice with subcutaneous B16-F10 tumors were treated twice inratumorally with 2.6x10^7^ ffu vMyx-IL15Ra-tdTr, showed a significant survival benefit for the treated group compared to the PBS control and the control virus (vMyx-tdTr). Fusion-protein expressing virus attenuated tumor growth and prolonged survival (median survival of 29, 23 and 17 days for vMyx-IL15Ra-tdTr, vMyx-tdTr and PBS treated groups, respectively). Immunohostological analysis of the subcutaneous tumors showed dramatically increased infiltration of NK cells in vMyx-IL15Ra-tdTr treated tumors compared to both controls. We hypothesized that the three virotherapeutic effects of the virus (oncolysis, delivery of IL15Rα-IL15, and immune activation from Toll like receptor-mediated inflammation) will augment the antitumor activities of host’s immune system. Our results suggest that IL15Rα-IL15 component does improve therapeutic effect over virus alone and that the effect is likely mediated by NK cell component of the immune system.

